# A new place for psychiatry among the forensic sciences

**DOI:** 10.1080/20961790.2021.1928815

**Published:** 2021-07-30

**Authors:** Godet Tony, Niveau Gérard

**Affiliations:** Geneva University Hospitals, Geneva, Switzerland

Forensic psychiatry occupies a special place among the forensic sciences. It has not always been recognized as a forensic science, and, even today, is not always integrated in forensic medicine centres in all countries. This may be because forensic psychiatry has not always demonstrated the rigour and robustness expected of a discipline that must be legally useful, and has thus faced criticism in the context of criminal trials.

Psychiatry originated as a medical discipline in the 18th century from alienism, which was primarily forensic medicine practice. Through physicians’ efforts to distinguish mentally ill people from delinquents within asylums, a branch of medicine called neuropsychiatry gradually emerged, followed by psychiatry. Forensic psychiatry has therefore existed for more than 200 years, but has long maintained an unclear identity that falls somewhere between psychiatry and forensic medicine. One reason for this ambiguity is the fact that forensic psychiatry has long combined, and often still combines, prison psychiatry. It is time to resolve this uncertainty, for both ethical and methodological reasons. From an ethical standpoint, prison psychiatry meets the classic criteria of medicine, as described by Beauchamp and Childress [1]; specifically, these include respect for patients’ autonomy, benevolence, non-malfeasance and fairness in the allocation of resources. Forensic psychiatry has a different ethical stance, in that it primarily aims for “the greatest good for the greatest number”. This is a consequentialist ethic, the central principle of which is impartiality [2].

Based on this clear ethical orientation, forensic psychiatry involves a methodology that is not one of individual care, but rather of service to the community. As such, forensic psychiatry must develop working methods and evaluation instruments that meet the rigorous standards of 21st century science. This special issue of *Forensic Sciences Research* represents the new willingness of forensic psychiatry to move in this direction and to be fully recognized within the forensic sciences.

The psychiatric evaluation of sexual assault statements made by minors is a good example of the ethics of neutrality that must prevail in all forensic psychiatry work. The value placed on children’s statements has varied greatly over time, ranging from total rejection, as in the Middle Ages, to total acceptance during the second half of the 20th century. The scientific analysis of such statements must extract itself from these *a ­priori* militants and focus on the search for objective credibility or non-credibility factors. Whilst computerized analysis of facial micro-expressions has made rapid progress, at present, the systematic study of the content of children’s statements remains the surest means of distinguishing between true and false declarations. An article by Niveau [3], which begins this special issue, reports the possibility that sensory information given by children during their court hearing could help to improve the precision of credibility evaluations.

The consequences of adverse childhood experiences represent an important area of forensic psychiatry. Whilst it has long been proposed that childhood trauma may be at the origin of multiple psychiatric and somatic illnesses during adulthood, recent research into epigenetic mechanisms has clarified the link between adverse childhood experiences and adult pathologies. In their systematic review, Neves et al. [4] examined studies on the relationship between adverse childhood experiences and DNA methylation. The authors found 19 studies that established a link between adverse childhood experiences and a predisposition to mental disturbances. From a scientific perspective, these results are of great importance for understanding the relationship between adult dyssocial behaviour and childhood traumatic experiences. In the years to come, epigenetics may be a factor to consider when assessing criminal responsibility.

Radicalization is a phenomenon of great interest in forensic science, mainly because of the consequences of terrorist acts. In forensic psychiatry, questions that arise concern not only the evaluation and care of survivors of terrorist acts, but also the evaluation of radi­calized individuals who must be judged in a court of law. General psychiatry is unprepared to confront this very unique phenomenon of radicalization. Garcet [5] has reported his direct experience with individuals in pre-trial detention who are suspected of terrorism. From these encounters, he developed a psychopathological model that provides a fuller understanding of the mechanisms of psychic functioning modification that are engaged during the radicalization process.

Understanding the social and psychopathological mechanisms underlying sexual delinquency is a constant interest of forensic psychiatry. Khoshnood et al. [6] have presented an innovative approach to these questions by analyzing a large sample of sexual aggressors using the Latent Class Analysis method. The authors identified two distinct profiles of aggressors—first-time offenders and repeat offenders. These criminological data constitute considerable progress for a fundamental question in forensic psychiatry, which is how to evaluate the risk of recidivism for this type of offender.

The perception of forensic psychiatry by the courts is often troubled by the uniquely clinical nature of the diagnostic procedures. The following two articles are therefore of great interest, as they explore the use of digital technologies in forensic psychiatry. Godet and Niveau [7] has tackled the problem of diagnosing the pathological sexual interests at the origin of acts of sexual delinquency against children. He reviewed eye-tracking studies and found that this technology, whilst still in its infancy, shows promise in the forensic domain.

Chang et al. [8] have reviewed the literature on the use of functional near-infrared spectroscopy in psychiatry. This technique is easy to use and has already been employed in numerous disciplines. In psychiatry, it makes it possible to identify cerebral dysfunctions associated with illnesses such as schizophrenia, bipolar disorder, and panic disorder. This imaging technology thus opens up a new field of research in forensic psychiatry, namely, the identification of cerebral mechanisms involved in the transition to pathological acts.

This special issue of *Forensic Sciences Research* was written during the COVID-19 pandemic. A contribution in this area was therefore expected. Jantzi and Perrin [9] describes a case of attempted suicide and altruistic infanticide following pandemic-induced psychiatric decompensation. She highlights the important relationship between forensic psychiatry and social contexts.

Through these rich and varied contributions, we hope that this special issue will contribute to a better integration of forensic psychiatry within the forensic sciences, and to a rigorous and innovative development of this speciality.

## Authors’ contributions

Tony Godet and Gérard Niveau wrote the draft and revised the manuscript. Both authors contributed to the final text and approved it.


Tony Godet
*Geneva University Hospitals, Geneva, Switzerland*


Gérard Niveau*Geneva University Hospitals, Geneva, Switzerland*Gerard.Niveau@hcuge.ch


